# Prediction of Protein–Protein Interaction Sites in Sequences and 3D Structures by Random Forests

**DOI:** 10.1371/journal.pcbi.1000278

**Published:** 2009-01-30

**Authors:** Mile Šikić, Sanja Tomić, Kristian Vlahoviček

**Affiliations:** 1 Department of Electronic Systems and Information Processing, Faculty of Electrical Engineering and Computing, University of Zagreb, Zagreb, Croatia; 2 Rudjer Bošković Institute, Zagreb, Croatia; 3 Bioinformatics Group, Department of Molecular Biology, Division of Biology, Faculty of Science, University of Zagreb, Zagreb, Croatia; Washington University, United States of America

## Abstract

Identifying interaction sites in proteins provides important clues to the function of a protein and is becoming increasingly relevant in topics such as systems biology and drug discovery. Although there are numerous papers on the prediction of interaction sites using information derived from structure, there are only a few case reports on the prediction of interaction residues based solely on protein sequence. Here, a sliding window approach is combined with the Random Forests method to predict protein interaction sites using (i) a combination of sequence- and structure-derived parameters and (ii) sequence information alone. For sequence-based prediction we achieved a precision of 84% with a 26% recall and an F-measure of 40%. When combined with structural information, the prediction performance increases to a precision of 76% and a recall of 38% with an F-measure of 51%. We also present an attempt to rationalize the sliding window size and demonstrate that a nine-residue window is the most suitable for predictor construction. Finally, we demonstrate the applicability of our prediction methods by modeling the Ras–Raf complex using predicted interaction sites as target binding interfaces. Our results suggest that it is possible to predict protein interaction sites with quite a high accuracy using only sequence information.

## Author Summary

In their active state, proteins—the workhorses of a living cell—need to have a defined 3D structure. The majority of functions in the living cell are performed through protein interactions that occur through specific, often unknown, residues on their surfaces. We can study protein interactions either qualitatively (interaction: yes/no) using large-scale, high-throughput experiments or determine specific interaction sites by using biophysical techniques, such as, for example, X-ray crystallography, that are much more laborious and yet unable to provide us with a complete interaction map within the cell. This paper presents the machine learning classification method termed “Random Forests” in its application to predicting interaction sites. We use interaction data from available experimental evidence to train the classifier and predict the interacting residues on proteins with unknown 3D structures. Using this approach, we are able to predict many more interactions in greater detail (i.e., to accurately predict most of the binding site) and with that to infer knowledge about the functions of unknown proteins.

## Introduction

Most proteins in a living cell interact in order to fulfil their function. Protein interactions occur through the formation of complexes, either transient or more long lasting, as a result of a balance between different molecular properties: sequence, shape, charge distribution, entropy and dynamics. Proteins often interact through multiple components, with examples like the replisome, RNA polymerases, the spliceosome, the ribosome, chaperonins and the various complexes formed along signal transduction pathways and during enzyme catalysis and inhibition. Knowledge of protein interactions is sometimes crucial in elucidating their functional roles. 3D structures of protein complexes have been the basis for detailed understanding of protein function; however, it is much more technically demanding to determine the structure of a complex as opposed to solving a structure of a single protein or even a fragment of the whole protein—a protein domain. This is the reason why in the current release of the Protein Data Bank (http://www.pdb.org) [1], 3D structures of protein complexes are poorly represented. In addition, the number of protein sequences deposited in the UniprotKB/Swiss-Prot database (http://www.uniprot.org) [2] outstrips the number of known 3D structures by around 7 times—a fact that further demonstrates the restricted effective size of the structural sample set available for studying protein interactions. On the other hand, experimental methods for detection of protein interaction residues from proteins without a known 3D structure are based on mutation and deletion studies. These methods are expensive, laborious and, most importantly, poorly applicable on a large scale.

The abundance of information that can be extracted from a 3D structure and sequence, the increase in computer power and the invention of novel classification methods have triggered development of computer based methods for prediction of protein interfaces. Since the pioneering work of Jones and Thornton [3] and their attempt to predict surface patches that overlap with interaction interfaces, several papers presenting different methods have been published. Methods presented therein can be roughly divided into three groups based on the choice of features used for prediction. The first group consists of methods based solely on sequence information that predict protein interfaces [4]–[8]. Methods in the second group [9]–[11] use structural information to refine sequence sets that are then used to construct predictors. Methods of the third group use 3D structure information exclusively or a combination of 3D structure and sequence for prediction [3], [12]–[17]. Selection of the classification method used also varies across different prediction tools: scoring functions [15], SVM (support vector machines) with radial kernel [6],[7] [9] [10] [14] and neural networks [5],[8],[11].

In this paper we present two methods for prediction of interaction sites of protein heterocomplexes using only a) sequence information; and b) information obtained from a combination of sequence and 3D structure features. Both new methods are based on the random forest algorithm [18] and linear classifier combinations. Our classification features are derived from sequence and spatial information, from sliding windows of nine residues in width. For the first time we rationalized this most commonly used window size through entropy analysis and demonstrated that it contains the highest amount structural information per sequence length.

Proteins commonly have many more residues that do not participate in an interaction than interacting residues, which creates an effect of imbalance between positive and negative datasets and must be dealt with in the process of classification. One drawback of imbalanced datasets is that some of the classification methods (especially the SVM) work with impaired performance and may introduce a bias in the resulting classification. Another consequence of working with an imbalanced dataset is that some commonly used evaluation measures, such as accuracy, are not appropriate because they favour the majority class [19]. Instead, we used the precision-recall graph, F-measure [20] and AUC (area under the ROC curve) [21], commonly used in the Information Retrieval sciences. It was demonstrated [22] that the classification method based on Random Forests achieves good results with unbalanced data. In addition we employed a classifier combination approach, which further improved predictions made from unbalanced data.

Performance comparison between different methods is rather difficult owing to the (i) lack of a good interaction benchmark set; (ii) different definitions of interaction sites; and (iii) different evaluation measures. Nevertheless, performance of our method in terms of structure-based prediction produces results comparable with best results obtained by other authors and we believe that our method outperforms others in the prediction of protein interacting residues based on sequence information alone.

For testing purposes, we built a Ras–Raf complex whose 3D structure has not been experimentally determined.

## Results

### The Length of a Sliding Window

**Figure 1:**
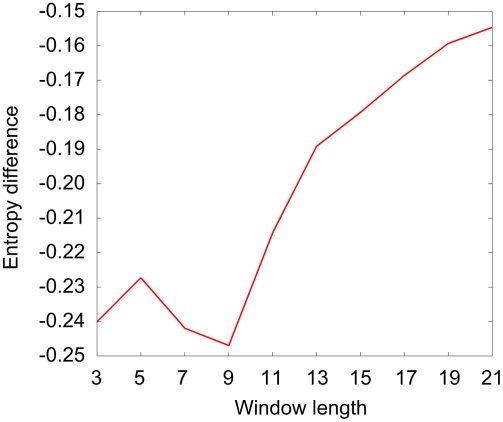
Entropy differences for different window lengths. Entropies for different window lengths were calculated and subtracted from entropies calculated for the uniform distribution of the number of interacting residues in the window.

The first step in our investigation was to determine the optimal sliding window length. We used a method (See Methods) based on the entropy difference between the occurrence of a particular number of interacting residues within a window length of *N* residues and the uniform occurrence distribution. We investigated only windows with a central interacting residue present. The result of the analysis is presented in Figure 1. Although the results for different window lengths are similar, it is evident that for the window length of 9 the entropy has the maximum difference.

### Prediction Using Sequence-Only Information

The most challenging part of our work was to construct a predictor of interacting residues using only sequence information. The input feature vector consisted of names of nine consecutive residues in a sequence. The class label of an instance was defined positive if at least *N* residues, including the central one, were marked as ‘interacting’. We classified data for values of *N* from 1 to 9. The evaluation of results is presented in Table 1. All of the presented values of measures, except the AUC, were calculated using a majority vote rule. The threshold for distinction between positive and negative output classes was 0.5. Table 2 shows the confusion matrix for a threshold of 1 interacting residue in a window. For error estimation 10-fold cross validation was used. It can be seen that the precision for almost all N's was over 80% with recall around 25%. When we further combined classifiers (See methods) the recall, F-measure and AUC increased, while the precision decreased. Using combining classifiers at a precision of 84%, we achieved a recall of about 26%. The F-measure, a harmonic mean of precision and recall obtained by combining classifiers was 40%, with the AUC at 74.7%. It is important to notice how accuracy increased as the ratio between positive labelled and negative labelled instances decreased. At the same time the precision was decreasing. If we further decreased the ratio between positive and negative labelled classes, accuracy would converge to the accuracy of the majority class, while precision would decrease to zero. Apparently, accuracy itself is not a good measure for evaluating method performance on an unbalanced dataset.

**Table 1:** Evaluation of the results obtained by prediction based on sequence information.

Threshold	Class Ratio^a^	Precision	Recall	F-measure	Accuracy	AUC
1	0.27	84.63	26.02	39.80	78.69	74.49
2	0.27	84.57	25.85	39.60	78.95	74.53
3	0.26	84.40	25.63	39.32	79.51	74.41
4	0.24	83.72	24.88	38.37	80.58	74.06
5	0.22	82.47	23.77	36.90	82.34	73.73
6	0.18	81.31	22.01	34.64	85.35	73.38
7	0.13	79.13	19.61	31.43	88.81	72.59
8	0.09	77.72	17.39	28.43	92.51	72.05
9	0.04	72.68	13.59	22.90	96.03	70.17
1 (combination)^b^	0.27	84.43	26.42	40.25	78.76	74.65

a The ratio between the number of positive labeled instaces and the number of negative labeled instances.

b The results obtained by combining classifiers.

**Table 2:** Evaluation of the results obtained by prediction based on sequence information–confusion matrix for threshold of 1 interacting residue.

	Actual Class = 0	Actual Class = 1
Predicted class = 0	121927	34097
Predicted class = 1	2178	11990

**Figure 2:**
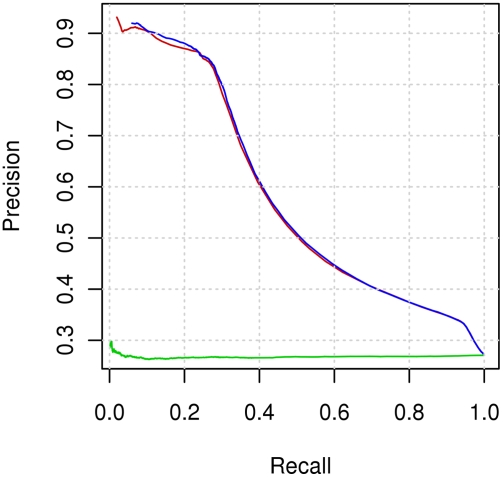
Precision–recall graph for prediction based on sequence alone. The figure presents precision–recall curves of the following methods: prediction of interacting residues when a positive class is labelled if at least the central residue is an interacting residue (red curve), combining classifiers (blue curve), randomization testing (green curve).

Figure 2 shows the precision-recall graph for combined classifiers. The results obtained by randomization testing (see Methods) are also presented. It can be seen that our method significantly outperforms random results.

In order to improve our results we introduced class weights. The Random Forests method uses different class weights for positive and negative classes in an effort to improve results of imbalanced data classification [22]. The results achieved using different weights are presented in Table 3. As can be noticed, the introduction of weights resulted in an increase in recall and F-measure, but with a decrease in precision. If we compare these values to those on the precision-recall graph it can be seen that the weighted classifiers are on or slightly above the curve. Random Forests is a discrete classifier so its output is represented with one point on the precision-recall curve. However, we can move along the curve to the desired values of precision or recall using different class weights.

**Table 3:** Evaluation of the results obtained by prediction based on sequence information using different class weights (threshold value is 1).

Weigh Ratio^a^	Precision	Recall	F-measure	Accuracy	AUC
1∶2	72.05	32.75	45.03	78.35	74.53
1∶3	48.17	52.83	50.39	71.83	74.42
1∶4	38.90	73.86	50.96	61.51	74.36
2∶3	82.34	28.15	41.95	78.91	74.51
2∶5	58.3	41.65	48.59	76.13	74.50

a Weights ratio between positive and negative labelled instances.

**Figure 3:**
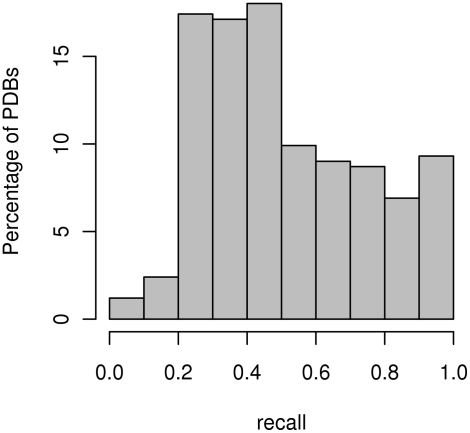
Histogram of recall values for protein complexes. The histogram of recall values for overall set precision at 48% and recall at 53%.

**Figure 4:**
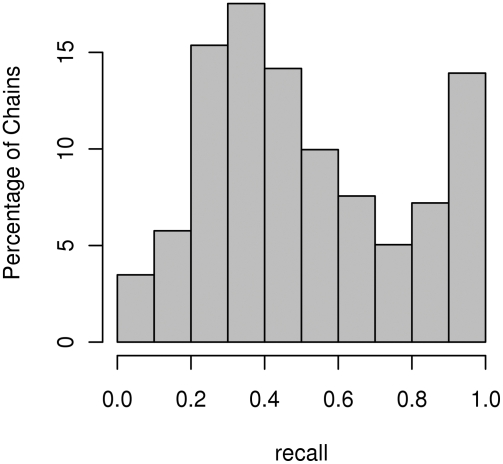
Histogram of recall values for chains. The histogram of recall values for overall set precision at 48% and recall at 53%.

Figures 3 and 4 show histograms of recall values for protein complexes and chains obtained for overall precision at 48% and recall at 53%. For these values our method correctly predicted at least one interaction site in 99.7% of the proteins and 99% of the chains.

For precision at 76% and recall at 31% we correctly predicted at least one interaction site in over 90% of the proteins and in over 80% of the chains.

### Predictions Obtained by a Combination of Sequence and 3D Structure Information

**Figure 5:**
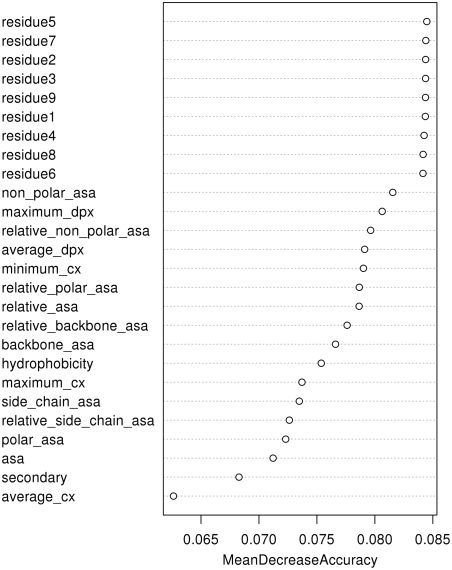
Variable importance. It can been seen that the most important variables are residue names, followed by non-polar ASA, maximum depth index, relative non-polar ASA, average depth index, and minimum protrusion index.

We analysed and used an exhaustive set of 3D structure based attributes (see Methods): accessible surface area (ASA) [23], depth index (DPX) [24], protrusion index (CX) [25], hydrophobicity as well as protein secondary structure. We used all 3D structure information available from PSAIA [26] with the addition of secondary structure. As the first step we performed training and prediction with all available sequence and 3D structure (a total of 26) features. The random forest algorithm has the capability to estimate the importance of a particular feature (an equivalent of the principal component analysis), so we employed it in the process of input parameter set reduction. Figure 5 shows the importance of particular features and their contribution to the overall prediction quality. It is evident that the information obtained from sequence has the highest importance. In addition, we also selected five best ranked structural features: non-polar ASA, maximum depth index, relative non-polar ASA, average DPX and minimum CX. With this reduced set of descriptors we obtained only slightly inferior results then by the entire dataset and therefore it was used in all subsequent analyses.

**Figure 6:**
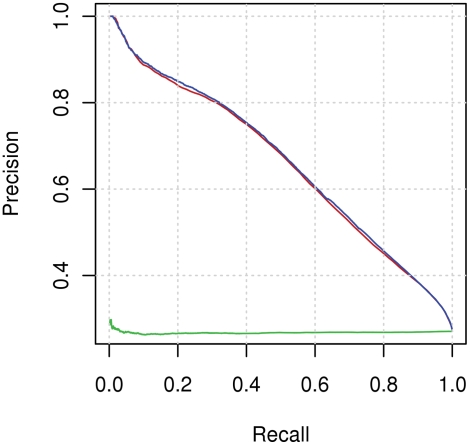
Precision–recall graph for prediction based on both sequence and 3D structure. The figure presents precision–recall curves of the following methods: prediction of interacting residues when a positive class is labelled if at least the central residue is an interacting residue (red curve), combining classifiers (blue curve), randomization testing (green curve).

We defined the central residue in the sliding window as an interacting residue if at least *N* of the residues (including the central residue) in the window are in contact with another chain. We tested threshold values of *N* in the range of 1 to 9. The evaluation of results is presented in Table 4. For precision at 78%, we achieved a recall of about 35%. When combining classifiers at the precision of 76%, we achieved a recall of about 38%. It can be seen that results obtained using combining classifiers are better. From the precision-recall graph (Figure 6) it is evident that prediction using structural information in combination with sequence information is better, especially in the central region, the most important part of the curve. Results with different class weights are presented in Table 5.

**Table 4:** Evaluation of the results obtained by prediction based on sequence and 3D structure information.

Threshold	Class Ratio^a^	Precision	Recall	F-measure	Accuracy	AUC
1	0.27	78.27	34.64	48.02	79.7	81.27
2	0.27	78.44	34.39	47.81	79.96	81.47
3	0.26	78.04	34.05	47.41	80.44	81.69
4	0.24	77.41	33.13	46.40	81.41	82.10
5	0.22	76.58	31.86	45.00	83.08	82.67
6	0.18	75.27	29.27	42.15	85.82	83.89
7	0.13	72.97	25.35	37.63	89.01	85.16
8	0.09	70.49	17.64	28.22	92.32	86.96
9	0.04	75.31	9.92	17.53	95.95	89.34
1 (combination)^b^	0.27	76.45	38.06	50.82	80.05	81.56

a The ratio between the number of positive labeled instaces and the number of negative labeled instances.

b The results obtained by combining classifiers.

**Table 5:** Evaluation of the results obtained by prediction based on sequence and 3D structure information using different class weights (threshold value is 1).

Weigh Ratio^a^	Precision	Recall	F-measure	Accuracy	AUC
1∶2	63.89	55.68	59.51	79.48	81.44
1∶3	52.54	70.08	60.05	74.76	81.42
1∶4	44.78	80.86	57.64	67.82	81.35
2∶3	71.68	45.68	55.8	80.40	81.46
2∶5	57.87	63.32	60.47	77.58	81.46

a Weights ratio between positive and negative labelled instances.

Similarly to predictions of interaction sites using only sequence information we evaluated the results per protein complex and chain. For precision at 75% and a recall of 40% our method correctly predicted at least one interaction site in over 97% of the proteins and over 90% of the chains. In addition for precision at 61% and a recall of 59% we correctly predicted at least one interaction site in 100% of the proteins and in over 99% of the chains.

### Ras–C-Raf

Reliability of our method was tested at the RBD (Ras Binding Domain) of C-Raf1 (PDB::1C1Y) and the wild type Ras (PDB::121P). Although the 3D structures of both C-Raf1 and Ras were solved, the structure of their complex has not been determined experimentally yet.

Using information from sequence and structure the following residues were predicted as potentially interacting: Ile21, Gln25, His27, Glu31, Asp33, Pro34, Thr35, Ile36, Glu37, Asp38, Ser39, Tyr40, Arg41, Lys42 and Ser65 (Ras protein) and Arg67, Val70, Val88, Glu104, Gly107, Lys108, Leu112 and Asp113 (C- Raf protein).

**Figure 7:**
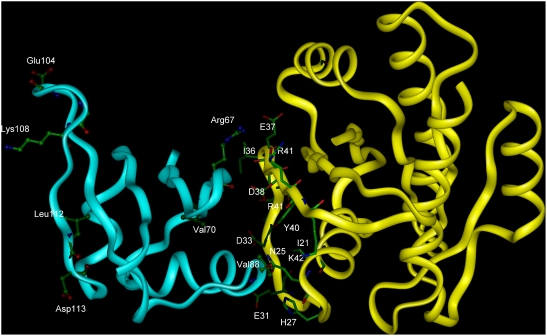
Model of the Raf–Ras complex. The amino acid residues found as possible interacting sites are labelled using the three letters type name for Raf and the one letter type name for Ras. The Ras residues are shown in stick representation, and Raf in ball and stick representation.

The complex was built by AutoDock, version 4.0, [27] using the Ras Protein as a receptor and by setting the centre of the grid to Ras Asp38, the central residue of the largest predicted interaction region. Docking simulations were carried out with an initial population of 200 individuals, and a maximum number of 2 500 000 energy evaluations. The model with the amino acids residues predicted as possible interacting sites labelled, is displayed in Figure 7. Majority of the predicted residues are part of the modeled complex interface and their importance for complex formation was found by the experiments [28]–[30] as well. Exceptions are Raf amino-acids Glu104, Lys108, Leu112 and Asp113 on the opposite side of its Ras binding interface. Although they have not been described in the literature as interacting residues, they might present interaction sites for some currently unknown interaction partner.

Stability of the complex was tested during 700 ps of molecular dynamics (MD) simulation performed by AMBER9 (http://amber.scripps.edu/) [31]. The proteins' conformations, as well as their mutual position, did not change significantly during the simulation: RMSD for the main chain atoms was 1.66 Å (RMSD for Raf was 1.31 Å, and for Ras 1.02 Å, final structure), and its plateau was achieved within 200 ps of unrestrained simulation, i.e., during the last 500 ps of simulations RMSD for the main chain was within 0.5 Å

## Discussion

### Comparison

**Figure 8:**
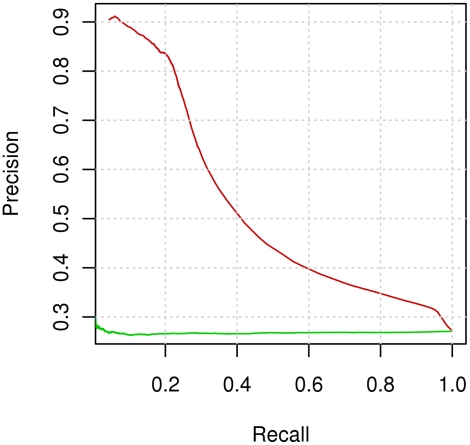
Precision–recall graph for prediction based on sequence alone and 3 –fold cross validation. The figure presents precision–recall curves of the following methods: prediction of interacting residues when a positive class is labelled if at least the central residue is an interacting residue (red curve) and randomization testing (green curve).

The main aim of this paper was to improve the prediction of interaction residues solely in protein sequence. In order to facilitate comparison, we used the same dataset and definitions of interacting sites as Ofran and Rost [5]. Because they divided sets of protein complexes into three subsets, we did the same for comparison of results. Figure 8 shows results obtained using 3-fold cross validation. For precision between 60 and 70%, Ofran and Rost achieved a recall of about 10% [8], while for the same precision we obtained a recall level of about 30%. The results can also be compared by the precision-recall (P-R) graphs where the P-R curve obtained by our methods shows better results, for recall at less than 50%. For higher recall values curves are similar. However, it is important to emphasise that the P-R curve obtained with randomization testing by our method has a lower value than theirs (27% compared to 30%). Because of that for recall values at more than 50% the curve obtained by our method is more distant from a random curve. Res et al. [7], did not present a precision-recall curve so we could compare only single point results. For the level of recall at 57.5% Res et al. [7] achieved a precision of 27.3%. This result is inferior to ours, i.e., for a recall of 57.5% we obtained precision above 40%.

Since authors [3], [13], [15]–[17] in the field of predicting interacting residues using 3D structure information used different estimation measures, datasets and definitions of interacting sites, it is difficult to objectively compare results.

### Usability

Docking of the Ras-Raf complex is an example of how our proposed methods can help with practical problems. Information on possible interacting residues can significantly help and speed up determination of reliable complex conformation. Similarly, prediction based on sequence information only, can help in the determination of possible deletion or mutation residues in experiments when 3D structure is unknown. Using different class weights a compromise can be made between expected prediction and recall of achieved results.

Finally, one of the results of this paper is the confirmation that a widow of nine concatenating residues contains the highest information content for prediction of interacting residues.

### Improvement

We believe that the results can be further improved in the ways explained below. For example, a bigger non redundant test set should be defined. The dataset we have used dates back to 2003. Since then, the number of experimentally determined 3D structures has increased from 20 000 to 50 000. In addition, methods proposed in this paper do not use information that other authors found valuable like evolutionary information [7],[8],[11],[16], electrostatics [13],[15] and desolvation [15]. Furthermore, it is evident that some 3D structure data like ASA improve prediction of interaction sites compared to sequence-only predictions. Hence, prediction of these 3D structure features from sequence through the usage of existing methods [32]–[34] or newly developed ones could further improve results.

Finally, the aggregation of interacting residues noticed by Ofran and Rost [5] might also be the beneficial approach. One way of using this information is described in the paper by Yan [9].

## Methods

### Dataset

For training and testing, we used a dataset of transient hetero interactions derived by Ofran and Rost [5]. The dataset consists of 1134 chains in 333 complexes. A residue was defined to be involved in a protein–protein interaction if any of its atoms were within 6 Å of any atom in a neighbouring non homologous chain. In our work, we used the PSAIA application [26] for the extraction of interacting residues. The main reason why we used the same dataset and the same method for definition of interacting residues as the above mentioned authors was for the purpose of comparing our results since their results are currently the best achievement in the field of proteins' interaction prediction from sequence alone.

### Input Features Vector

The input vector of features was defined on a sliding window of nine residues. The window was defined as positive, if the central residue and at least *N*−1 other residues were interacting residues. We used a value for *N* in a range of 1 to 9. For determination of true negatives we used a method similar to the one of Ofran and Rost. We made an alignment of all homologous chains (at least 90 percent of sequence similarity) in the 3D structure of a complex. If all aligned chains at a particular site had the same nine residues in the window and none of them had a central residue in contact with a neighbouring non homologous chain we defined this window as a true negative.

The input vector consists of nine residues' names, and min, max or average values for features that belong to residues in the window. In this paper we used the following features:

- Secondary structure of the central residue
- Average hydrophobicity [35]
- Average ASA (accessible surface area)
- Average relative ASA
- Average backbone ASA
- Average relative backbone ASA
- Average backbone ASA
- Average relative backbone ASA
- Average non-polar ASA
- Average relative non-polar ASA
- Average polar ASA
- Average relative polar ASA
- Average depth index (DPX)
- Average protrusion index (CX)
- Minimal protrusion index
- Maximal protrusion index [25]
- Maximal depth index [24]

The average value of a particular feature k was calculated as:
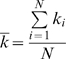
where *i* was the ordered number of a residue in the window of *N* = 9 residues. The secondary structure information was extracted by DSSP [36], while for extraction of all other features PSAIA was used [26].

### Length of the Sliding Window

The length of the sliding window can influence the classification of results. For determination we used a method based on entropy. First we defined the interacting residues for all proteins in the datasets. Secondly, we calculated the number of interacting residues using sliding windows of different lengths. Only the windows with a central interacting residue were taken in consideration. Finally, the entropies for different window lengths were calculated, and subtracted from entropies calculated for a uniform distribution of numbers of interacting residues in the window. As the best result we defined the one with a highest calculated entropy difference. The calculation can be shown as following:
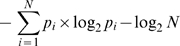
where *N* is the length of a window, *p_i_* is the frequency appearance of *i* interacting residues in a window of *N* residues, given a central interacting residue.

The uniform distribution of a particular set has the highest entropy, so data that has the highest difference from that value has more structure than others and it is easiest to describe.

### Measuring Performance

The results reported in this paper concern the evaluation of residue classification based on the following quantities: the number of true positives (TP) (residues correctly classified as interacting), the number of true negatives (TN) (residues correctly classified as non-interacting), the number of false positives (FP) (non-interacting residues incorrectly classified as interacting), and the number of false negatives (FN) (interacting residues incorrectly classified as non-interacting). These values are usually presented in a confusion matrix. We use the following measures of performance:
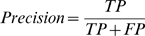


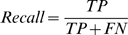


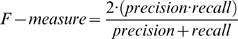


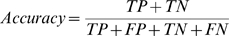
In addition we used a precision-recall graph and area under the ROC curve (AUC) [21] for comparison of the results of our method with a random classifier. Although we believe that accuracy is not an appropriate measure in the event of imbalanced data we used it as a directly comparable measure to results of other prediction methods. In Random Forests there is no need for cross-validation or a separate test set to get an unbiased estimate of the test set error. It is estimated internally with the out of bag error estimate [18]. When estimating the test error using both methods we achieved a very slight difference in results, but for comparison to other results we present results obtained by 10-fold cross validation. For cross validation we divided a set of 333 proteins into 10 subsets, so that we would not use data from the same proteins in test and training sets. Performances of classifiers were estimated by the ROCR R package [37].

### Randomisation Testing

Classification methods are sensitive to over-fitting so it was important to measure the significance of obtained AUC values and precision-recall graphs. Randomisation testing has been found to be very effective at assessing over-fitting [38],[39]. Here, the original training set was copied and class labels were replaced with random class labels. The ratio between positive and negative class labels was preserved. Then the Random Forests were trained with these data using the same methodology that was used with the original data.

### Random Forests

Random Forests [18] is an ensemble method that combines several individual classification trees in the following way: from the original sample several bootstrap samples are drawn, and an unpruned classification tree is fitted to each bootstrap sample. The feature selection for each split in the classification tree is conducted from a small random subset of predictor variables (features). From the complete forest the status of the response variable is predicted as an average or majority vote of the predictions of all trees. Random Forests is often used when we have very large training datasets and a very large number of input features (hundreds or even thousands of input features). A random forest model is typically made up of tens or hundreds of decision trees. In this paper we used 200 trees. As part of the algorithm, Random Forests returns few measures of feature importance. The most reliable measure is based on the decrease of classification accuracy when values of a feature in a node of a tree are permuted randomly and this is the measure of feature importance that we used in this paper. PARF (parallel Random Forests) [40] implementation of the random forest method and the randomForest R package [41] were used for classification.

Random Forest method is a discrete classifier. When such a classifier is applied to a test set, it yields a single confusion matrix, which in turn corresponds to a single point on a ROC curve. However, it is possible to use, as output, the percentage of votes for a particular class. Using different threshold values for producing positive or negative response variables it is possible to produce ROC and precision-recall curves.

### Combining Classifiers

A simple method for combining classifiers was used in this paper. The method takes output of the first stage classifiers as input values for the second stage. The output of the second stage is a positive class if at least one of the input values was positive. This method achieves good results with imbalanced data (Šikić and Jeren, manuscript in preparation). In this paper we labelled a sliding window instance with a positive class if it contained equal or more interacting residues than a value defined by the threshold and if the central residue was an interacting residue. We made classifications with threshold values from 1 to 9. The outputs of all of these classifiers were combined as explained above.

To better describe this method let us take one example. We start the prediction process by selecting a sliding window of nine residues. First we make a prediction using the classifier for threshold 1. This classifier is trained to predict interaction sites if at least the central residue is in an interaction. Second we make a prediction using the classifier for threshold 2. This classifier is trained to predict interaction sites if the central residue and at least one other residue inside the window are in an interaction. Using same method we make a prediction for thresholds 3 to 9. It can be easily seen that instances labelled positive (windows) for classifiers that use thresholds 2 to 9 are subsets of positive instances for classifiers that use threshold one. If we assume that it is possible that some classifiers can in some cases more accurately predict subsets than the original set we can combine classifiers using the OR rule. Hence, the output class label would be positive if at least one classifier labelled that instance as positive.
